# DNA methylation: potential biomarker in Hepatocellular Carcinoma

**DOI:** 10.1186/2050-7771-2-5

**Published:** 2014-03-17

**Authors:** Way-Champ Mah, Caroline GL Lee

**Affiliations:** 1 Department of Biochemistry, Yong Loo Lin School of Medicine, National University of Singapore, Singapore 117597, Singapore; 2 Division of Medical Sciences, Humphrey Oei Institute of Cancer Research, National Cancer Centre Singapore, Level 6, Lab 5, 11 Hospital Drive, Singapore 169610, Singapore; 3 NUS Graduate School for Integrative Sciences and Engineering, National University of Singapore, Singapore 117456, Singapore; 4 Duke-NUS Graduate Medical School, Singapore 169857, Singapore

**Keywords:** Epigenetics, Methylation, Biomarker, CIMP, Hepatocellular carcinoma, Prognosis, Diagnosis

## Abstract

Hepatocellular Carcinoma (HCC) is one of the most common cancers in the world and it is often associated with poor prognosis. Liver transplantation and resection are two currently available curative therapies. However, most patients cannot be treated with such therapies due to late diagnosis. This underscores the urgent need to identify potential markers that ensure early diagnosis of HCC. As more evidences are suggesting that epigenetic changes contribute hepatocarcinogenesis, DNA methylation was poised as one promising biomarker. Indeed, genome wide profiling reveals that aberrant methylation is frequent event in HCC. Many studies showed that differentially methylated genes and CpG island methylator phenotype (CIMP) status in HCC were associated with clinicopathological data. Some commonly studied hypermethylated genes include p16, SOCS1, GSTP1 and CDH1. In addition, studies have also revealed that methylation markers could be detected in patient blood samples and associated with poor prognosis of the disease. Undeniably, increasing number of methylation markers are being discovered through high throughput genome wide data in recent years. Proper and systematic validation of these candidate markers in prospective cohort is required so that their actual prognostication and surveillance value could be accurately determined. It is hope that in near future, methylation marker could be translate into clinical use, where patients at risk could be diagnosed early and that the progression of disease could be more correctly assessed.

## Introduction

Hepatocellular Carcinoma (HCC) is one of the most frequent cancers in the world and annually, about 600,000 patients died of liver cancer [1]. This disease is often associated with poor prognosis because patients are either diagnosed at very late stage or experienced recurrence after resection [2]. In fact, more than half of HCC patients died within 12 months post diagnosis, and less than 6% of them have an average survival rate of 5 years [3]. Liver transplantation and resection are the only two curative therapies available; however, in order to qualify for such therapies, patients need to be diagnosed early with HCC [4]. Presently, serum alpha-fetoprotein (AFP) concentration and hepatic ultrasonography are used in HCC surveillance program, where high risk patients are screened for HCC in every six months [5]. As for actual diagnosis, invasive biopsy and expensive imaging tools such as ultrasonography, spiral computed tomography (CT) and magnetic resonance imaging (MRI) are used [4]. AFP measurement is merely used as adjunct diagnostic tool because of its variability in specificity and sensitivity [5,6]. Equally important to note is that apart from AFP level and tumor staging classification such as the Barcelona Clinic Liver Cancer (BCLC) staging system, there is no good prognostic marker that can classify patients and predict survival outcome [7-9]. The large number of HCC associated deaths clearly reflects the shortcomings of current diagnostic and prognostic tools. This underscores the importance of discovering novel and effective biomarkers that can improve overall clinical management of HCC.

With the advance of genomic technologies, plethora of molecular data is now available for translational research. Gene expression signatures and microRNA profiles are just some examples of molecular data that were actively explored as potential biomarkers for HCC [10-15]. In this review, we will focus specifically on DNA methylation, another potential biomarker that was shown to be implicated in HCC.

## Review

### Aberrant DNA methylation in HCC

**Figure 1:**
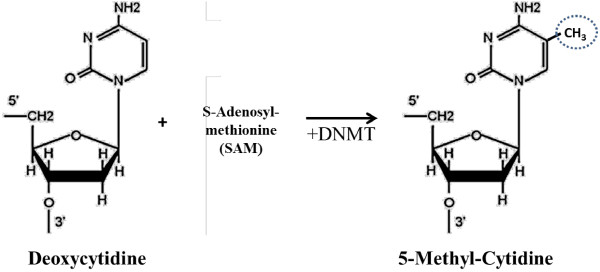
**Structures of deoxycytidine and 5-methyl-deoxycytidine.** DNA methylation occurs when a methyl group is attached to the 5^th^ carbon of cytosine, where DNMT serves as enzyme and SAM acts as the methyl group donor.

Studies have identified a few somatic mutations in HCC, for instance, mutations in TP53 [16,17], CTNNB1 [18,19], and AXIN1 [20,21]. However, frequencies of these mutations are inconsistent and rare, some occur only in certain subtypes of tumor [22]. The lack of common genetic marker associated with HCC cases strongly suggests that epigenetic alterations such as aberrant DNA methylation could be the alternative factor contributing towards liver carcinogenesis. DNA methylation occurs when a methyl group is attached to the 5^th^ carbon of cytosine nucleotide and this process is catalyzed by DNA methyltransferases (DNMTs), in which S-adenosyl-methionine (SAM) acts as a methyl donor (Figure 1) [23]. Deregulation of DNA methylation was shown to be associated with many cancers, as was first proposed by Andrew Feinberg and Bert Vogelstein in 1983 [24]. The two most common forms of aberrant methylation are global hypomethylation and site-specific hypermethylation. In HCC, such deregulations are frequently observed as well. Global hypomethylation in liver cancer affects the structural-nuclear function by promoting chromosomal and genomic instability, while regional hypermethylation is often associated with silencing of tumor suppressor genes [25]. Studies have revealed that etiological factors like Hepatitis virus infection may lead to aberrant DNA methylation in cancerous tissues [26,27]. DNA methyltransferases such as DNMT1, DNMT3A and DNMT3B were also shown to be up-regulated in liver cancer [28,29]. Whether increased expression of DNMTs associated with aberrant methylation of genes is still a matter of controversy as the exact mechanism has yet to be elucidated [29,30]. Subsequent sections will summarize respective studies on aberrant DNA methylation in hepatocarcinogenesis and the full list of studies can be found in Additional file 1: Table S1.

### Genome wide studies on methylation profile of HCC

Present technologies allow researchers to profile methylation in a genome wide manner. Two commonly used methods in HCC methylation profiling include hybridization of bisulfite converted DNA on beadarray [31,32] and enrichment of methylated DNA either by enzymatic digestion [33,34] or antibody pull-down [35], followed by promoter array profiling. Even though high throughput sequencing is becoming more available, presently, no study has yet to use this approach to map the methylome of HCC. Table 1 briefly summarizes the strengths and limitations of each profiling method. A few pivotal genome wide methylation studies using these approaches are highlighted below.

**Table 1:** Different methods in genome-wide methylation profiling

	**Platform**	**Features**	**Number of regions analysed per sample**	**Methylation information on site specific CpG loci**	**Methylation information on non-CpG loci**	**Advantages**	**Disadvantages**
**Microarray based**	Methylated CpG Island Amplification and Microarray (MCAM-chip)	Enzyme-based techniques that rely on restriction enzymes (SmaI and XmaI) followed by profiling on promoter array	~25,000 human promoters (depends on array density)	No	No	Do not require bisulfite conversion, good coverage on region with low CpG density.	Require substantial quantities of input genomic DNA, low sample throughput, do not report methylation status at single nucleotide level, bias may occur due to genomic distribution of CpG loci, limited to mostly promoter regions.
	Differential Methylation Hybridization and Microarray (DMH-chip)	Enzyme-based techniques that rely on restriction enzymes (MseI and BstUI) followed by profiling on promoter array					
	Methylated DNA Immunoprecipitation and Microarray (MeDIP-chip)	Immunoprecipitation of methylated DNA with a monoclonal antibody followed by profiling on promoter array					
**Beadarray based**	GoldenGate	Bisulfite convertion of DNA followed by microbead based microarray	~1,500 CpG sites	Yes	No	Require minimum input genomic DNA, high sample throughput, provide methylation status at CpG loci, fairly accurate and reproducible.	Bisulfite treatment may not be complete, bisulfite treatment caused DNA degradation, limited to mostly promoter regions.
	Infinium 27K		~27,000 CpG sites					
	Infinium 450K		~450,000 CpG sites					
**High throughput sequencing**	Bisulfite sequencing	Bisulphite conversion of DNA followed by capture and high throughput sequencing	Whole genome	Yes	Yes	High resolution mapping of methylation status at single nucleotide level, no cross hybridization bias.	Bisulfite treatment may not be complete, bisulfite treatment caused DNA degradation, low sample throughput, expensive, complex bioinformatic analysis.	

In 2008, Gao et al. adopted methylated CpG island amplification microarray (MCAM) method to identify 719 genes that were differentially methylated between tumors and adjacent non-tumors [36]. They used pyrosequencing to validate their observations found by MCAM. Genes such as RASSF1A, CDKN2A and CCNA1 were successfully validated to be highly methylated in cancer tissue compared to adjacent non-tumor and normal liver tissues. In subsequent year, Lu et al. used differential methylation hybridization (DMH) method to locate 38 hypomethylated and 27 hypermethylated regions. Using Methylation specific PCR (MSP) method, they validated the methylation status of KLK10 and OXGR1 in tumors, and found that hypermethylation of KLK10 was associated with Hepatitis C virus (HCV) infection and cirrhosis [37]. Around the same time, studies by Deng et al. and Stefanska et al. used a slightly different method called methylated DNA immunoprecipitation microarray (MeDIP-chip) to locate aberrant methylation in HCC. Deng et al. used MassArray® method to validate hypermethylation of DUSP4, NPR1 and CYP24A1 in HCC, and correlate methylation status of these genes with recurrence free survival [38]. Stefanska et al., on the other hand, delineated the profile of promoter hypomethylation in HCC and validated AKR1B10, CENPH, MMP9, MMP12, PAGE4, S100A5, MMP2 and NUPR1 to be hypomethylated in liver cancer using pyrosequencing [39]. Earlier genome wide studies utilised promoter microarray to map out differentially methylated regions. As a result, such arrays could not provide information on site specific CpG dinucleotides that were aberrantly methylated. Additional validation steps such as pyrosequencing and MassArray® were required before one could locate the exact deregulated CpG sites. Nonetheless, as technology of methylation profiling matures over recent years, many studies could now report genome wide methylation status of HCC at single-nucleotide resolution (Table 2). These studies mainly used the beadarray technology developed by Illumina®. As shown in Table 2, the most recent studies by Song et al., Zhang et al. and Shen et al. reported the mapping of more than 485000 CpG sites, the highest throughput so far, in HCC.

**Table 2:** Genome wide methylation profiling in HCC

**Promoter microarray**					
Discovery method	HCC patients (n)	Validation method	Validated genes	Publication	Year
MCAM-chip	10	Pyrosequencing	RASSF1A, p16, TBX4, MMP14, GNA14, SLC16A5, CCNA1	Gao et al. [36]	2008
	16	Pyrosequencing	KLHL35, PAX5, PENK, SPDYA, LINE-1	Shitani et al. [40]	2012
DMH-chip	21	MSP	KLK10, OXGR1	Lu et al. [37]	2008
MeDIP-chip	6	MassArray	CYP24A1, DLX1, ZNF141, RASGRF2, ZNF382, TUBB6, NPR1, RRAD, RUNX3, LOX, JAKMIP1, SFRP4, DUSP4, PARQ8, CYP7B1	Deng et al. [38]	2010
	11	Pyrosequencing	AKR1B10, CENPH, MMP9, MMP12, PAGE4, S100A5, MMP2, NUPR1	Stefanska et al. [39]	2011
**Beadarray**					
Discovery method	HCC patients (n)	Validation method	Validated genes	Publication	Year
GoldenGate	20	Methylight assay	APC	Archer et al. [49]	2010
	38	Pyrosequencing	RASSF1, GSTP1, APC, GABRA5, LINE-1	Hernandez-Vargas et al. [43]	2010
	45	Bisulfite sequencing	ERG, HOXA9	Hou et al. [47]	2013
Infinium 27K	3	COBRA and bisulfite sequencing	WNK2, EMILIN2, TLX3, TM6SF1, TRIM58, HIST1H4F, GRASP	Tao et al. [44]	2011
	62	NIL	NIL	Yang et al. [118]	2011
	13	NIL	NIL	Ammerpohl et al. [48]	2012
	62	Pyrosequencing	CDKL2, STEAP4, HIST1H3G, CDKN2A, ZNF154	Shen et al. [42]	2012
	63	Pyrosequencing	PER3	Neumann et al. [50]	2012
	71	Pyrosequencing	NEFH, SMPD3	Revill et al. [53]	2013
Infinium 450K	66	NIL	NIL	Shen et al. [46]	2013
	27	Pyrosequencing	GSTP1, RASSF1, BMP4, DLGAP1, GPR35	Song et al. [45]	2013
	6	Bisulfite sequencing	DBX2, THY1	Zhang et.al. [41]	2013

*COBRA*, Combined bisulfite restriction analysis; *MSP*, Methylation specific PCR.

Genome wide methylation profiling provides wealth of information for downstream analysis. Using these high throughput data, researchers could efficiently separate tumors from adjacent non-tumors [39-47], cirrhotic liver from HCC [48,49], and cluster the tumors according to their risk factors such as viral infection [38,43,46] and alcohol consumption [42,43]. Novel tumor suppressor genes that were silenced by methylation could also be uncovered through genome wide studies, as shown by studies in [37,50-53]. As reports on genome wide methylation profile of HCC patients using serum DNA begin to emerge [41,42], it is hoped that these high throughput data could accelerate the process of biomarker discovery.

### Methylation as prognostic marker

From 2003 to 2013, many studies have published on the prognostic values of DNA methylation in HCC. These studies are summarized in Additional file 1: Table S1. Due to space constraints, within this review, only a few examples will be highlighted. One of the early studies was reported by Yang et al. Their group profiled methylation status of 9 genes, namely GSTP1, SOCS1, CDH1, APC, p15, p16, p14, p73 and RAR-β in 51 HCC samples using methylation-specific polymerase chain reaction (MSP) [54]. Among these genes, methylation of SOCS1, APC and p15 were shown to be more frequently observed in HCV-positive HCC patients compared to HBV/HCV-negative HCC. Another group from Korea, Lee et al. examined the methylation level of CpG loci in 14 genes in sixty HCC paired samples and found that methylation of GSTP1 and CDH1 were associated with poorer overall survival [55]. Similarly, Yu et al. used MSP to identify methylation level of 24 genes in 28 HCC samples from a Chinese population. They successfully showed that methylation of AR, DBCCR1, IRF7, OCT6, p73, and p16 were associated with late stage HCC [56]. These three studies laid a strong basis for subsequent methylation analysis. Many studies have since then attempted to associate clinical parameters with DNA methylation, particularly on genes that were validated in these three studies. Subsequent paragraphs will outline 4 of these genes, namely, p16, SOCS1, GSTP1 and CDH1 (Table 3).

**Table 3:** Commonly studied methylation markers in HCC

**Gene**	**HCC patients (n)**	**Clinicopathological correlation**	**Validation method**	**Publication**	**Year**
**p16**	28	Tumor stage	MSP	Yu et al. [56]	2003
	18	Tumor stage	MSP	Shim et al. [59]	2003
	20	Tumor differentiation	MSP	Qin et al. [66]	2004
	50	Age, gender, virus infection (HBV/HCV)	MSP	Li et al. [62]	2004
	44	HBV infection	MSP	Jicai et al. [63]	2006
	60	Age, tumor stage, vascular invasion, virus infection (HBV/HCV)	MSP	Katoh et al. [61]	2006
	58	Tumor stage	MSP	Su et al. [60]	2007
	23	HBV infection (HBx)	MSP	Zhu et al. [65]	2007
	265	Disease free survival	MSP	Ko et al. [70]	2008
	118	Gender	Methylscreen	Wang et al. [119]	2012
**SOCS1**	50	Liver cirrhosis	MSP	Okochi et al. [77]	2003
	51	HCV infection	MSP	Yang et al. [54]	2003
	284	Age, tumor size, virus infection (HBV/HCV)	MSP	Ko et al. [73]	2008
	77	HCV infection	COBRA	Nishida et al. [76]	2008
	46	Liver cirrhosis, tumor size	MSP	Chu et al. [75]	2010
	46	Tumor stage	MethyLight	Um et al. [72]	2011
	29	Chemotherapy treatment	MSP	Saelee et al. [120]	2012
	116	Age and gender	Methylscreen	Zhang et al. [74]	2013
**GSTP1**	60	Overall survival	MSP	Lee et al. [55]	2003
	83	Alcohol consumption, gender	MSP	Zhang et al. [82]	2005
	60	Gender, viral infection (HBV/HCV)	MSP	Katoh et al. [61]	2006
	58	HBV infection, tumor stage	MSP	Su et al. [60]	2007
	77	HCV infection	COBRA	Nishida et al. [76]	2008
	166	HBV infection	Pyrosequencing	Lambert et al. [81]	2011
**CDH1**	60	Overall survival	MSP	Lee et al. [55]	2003
	32	Vascular invasion, recurrence	MSP	Ghee [91]	2005

*COBRA*, Combined Bisulfite Restriction Analysis; *HBV*, Hepatitis B virus; *HCV*, Hepatitis C virus; *HBx*, Hepatitis B virus X protein; *MSP*, Methylation-specific PCR.

### Commonly studied methylation marker genes

p16 (CDKN2A) is one of the most reported genes that was shown to be hypermethylated and associated with clinical parameters in HCC. It is a tumor suppressor gene that plays a role in cell cycle regulation [57]. It was methylated in many other cancers as well [58]. Beside earlier study by Yu et al. [56], Shim et al. [59] and Su et al. [60] also reported that methylation level of p16 was associated with advanced stage of HCC. They showed that methylation of p16 gene increased from cirrhotic tissue to HCC. Studies have also shown that hepatitis virus positive HCC samples have higher p16 methylation compared to HCC with no viral infection [61-64]. Zhu et al. even further showed that HBx gene, a protein coded by HBV, was associated with methylation of p16 in HBV positive HCC samples [65]. Clearly, environment factors such as viral infection could possibly disturb the epigenetic profile of the liver and contribute towards carcinogenesis. In addition, vascular invasion [61] and tumor differentiation [66] were also shown to be associated with p16 methylation. As vascular invasiveness and tumor differentiation were both strong predictors of survival in HCC [67-69], it is not surprising that hypermethylation of p16 in HCC patients was shown to have worse disease free survival as well [70].

Another frequently studied prognostic marker is SOCS1 methylation. SOCS1 gene was shown to be negative regulator of JAK/STAT pathway and its suppression by hypermethylation promotes cell growth [71]. SOCS1 methylation was correlated with progression of HCC [72], age [73,74] and tumor size [73,75]. Moreover, as mentioned in earlier paragraph, SOCS1 methylation was shown by Yang et al. to be associated with HCV infected HCC [54]. Concurring their study, Nishida et al. [76] and Ko et al. [73] also revealed that SOCS1 methylation was more prevalent in HCV infected HCC compared to non-infected HCC. Interestingly, Chu et al. [75] and Okochi et al. [77] did not find this association to be significant in their studies; instead, they found liver cirrhosis in HCC to be closely linked to hypermethylation of SOCS1. As HCV infection may lead to liver cirrhosis [78], more studies are required to ascertain this pathological link between HCV infection, SOCS1 methylation and HCC progression.

GSTP1 belongs to Glutathione S-transferases family, where it plays a role in protecting cells against damage induced by carcinogens, and modulating signal transduction pathways that control cell proliferation and cell death [79]. Promoter methylation of GSTP1 was first reported in prostatic carcinoma back in 1994 [80]. Since then, many groups reported such observation in other cancers, including HCC. Analogous to earlier mentioned two genes, GSTP1 was also found to be highly methylated in HCC infected with either HBV or HCV compared to non-infected HCC [60,61,76,81]. Interestingly, methylation of GSTP1 was significantly associated with gender [61,82] and alcohol intake [82]. Also, study by Lee et al. managed to show that patients with high GSTP1 methylation level have worse overall survival outcome [55]. Although many studies examined the association of GSTP1 methylation with clinicopathological characteristics, only a few found associations suggesting that GSTP1 methylation alone may not be sufficient to serve as good single prognostic predictor for HCC.

CDH1 is another well-known tumor suppressor gene that was found methylated in many cancers [83-88]. It was frequently methylated in HCC as well. Despite many studies showed that methylation of CDH1 was higher in HCC than adjacent non-tumors, it often was not significantly associated with clinical parameters [54,61,76,89,90]. Only Lee et al. reported that methylation of CDH1 was linked with worse overall survival [55], and Ghee et al. found that it was associated with vascular invasion and recurrence [91]. These reports suggest that probably CDH1 alone may not have the power to be an independent prognostic factor. Notably, the concurrent methylation of CDH1, GSTP1 and a few other genes were found to be significantly associated with levels of AFP, recurrence free survival (RFS) and tumor numbers (Table 4). This concordant methylation of a group of genes associated with specific tumor characteristics is known as CIMP or CpG island methylator phenotype [92]. Presently, many studies have attempted to elucidate CIMP in various cancers, including G-CIMP for gliomas [93], B-CIMP for breast cancer [94], and C-CIMP for colorectal cancer [95]. In HCC, concurrent methylation of various genes has been associated with various clinical phenotypes (Table 4) and these could perhaps be known as CpG island methylator phenotype for hepatocellular carcinoma, or Hep-CIMP.

**Table 4:** CIMP studies in HCC

**Genes used to define CIMP**	**HCC patients (n)**	**Clinicopathological correlation**	**Validation method**	**Publication**	**Year**
ER, c-MYC, p14, p15, p16, p53, RB1, RASSF1A, WT1	50	AFP level	MSP	Zhang et al. [96]	2007
E2F1, p15, p16, p21, p27, p300, p53, RB, WT1	120	Metastasis	MSP	Zhang et al. [97]	2008
CDH1, p14, p15, p16, p21, RB1, RASSF1A, SYK, TIMP3, WT1	60	Metastasis, tumor stage	MSP	Cheng et al. [98]	2010
CDH1, DAPK, GSTP1, p16, SOCS1, SYK, XAF1	65	AFP, RFS, tumor numbers	MSP	Wu et al. [100]	2010
GSTP1, MGMT, OPCML, p14, p15, p16, p73, RARβ, SOCS1	115	OS, RFS	MSP	Li et al. [99]	2010
APC, CDH1, DKK, DLC1, RUNX3, SFRP1, WIF1	108	AFP level, DFS, Gender, HBV status, tumor stage	MSP	Liu et al. [101]	2011
APC, GSTP1, HIC1, p16, PRDM2, RASSF1A, RUNX3, SOCS1	177	DFS	Methylight	Nishida et al. [102]	2012

*AFP*, Alpha fetoprotein; *DFS*, Disease free survival; *HBV*, Hepatitis B virus; *MSP*, Methylation-specific PCR; *OS*, Overall survival; *RFS*, Recurrence free survival.

### Hep-CIMP associated with clinicopathological parameters

Presently, almost all studies that attempt to characterize Hep-CIMP came from Chinese population and methylation level of these CIMP genes were determined by MSP method. L-X, Wei led a team that published three studies on Hep-CIMP from 2007 to 2010. They defined CIMP + as samples with five or more methylated marker genes. Interestingly, marker genes that they used to define CIMP status varied across three studies (Table 4). Nonetheless, they managed to associate CIMP status with elevated AFP level (AFP ≥ 30 μg/L) [96], tumor metastasis [97,98], telomerase activity [97], tumor–node–metastasis (TNM) staging and overall survival [98].

Li et al. on the other hand, examined the methylation status of nine marker genes, namely, p14, p15, p16, p73, GSTP1, MGMT, RARβ, SOCS-1, and OPCML in 115 tumors. They defined CIMP + as HCC samples with six or more such methylated genes. They showed that CIMP + patients with TNM stage I have significantly poorer recurrence-free survival (RFS) and overall survival (OS) compared to CIMP-, TNM stage I patients [99]. Wu et al. also reported similar observation, despite their definition of CIMP + differed slightly [100]. They considered a sample to be CIMP + as long as it has three or more methylated marker genes. Their study revealed that CIMP + patients have shorter RFS compared to CIMP- patients. On top of that, they also showed that tumor number and pre-operative AFP levels were significantly higher in CIMP + samples [100].

More recently, Liu et al. reported the CIMP status of 108 HCC tissues and plasma respectively, based on methylation level of seven marker genes (Table 4). They found good concordance of methylation status between plasma and tissue samples. CIMP status in tumor tissues and plasma were both significantly associated with clinicopathological parameters such as AFP level, TNM staging, gender and HBV infection [101]. Lastly, Nishida et al. profiled methylation status of eight genes, namely, HIC1, SOCS1, GSTP1, p16, APC, RASSF1, PRDM2 and RUNX3, and found that these markers, collectively, were associated with shorter time-to-occurrence of HCC tumor [102]. Even though Nishida et al. did not report CIMP status in their study, their analysis was similar to the rest of the Hep-CIMP studies. Also worthy to note is that these markers were carefully selected to represent very early stage of HCC. This again emphasizes the potential clinical use of early CIMP + signature for diagnosis or prognosis purposes.

Currently, there is still no consensus on how we define CIMP for HCC. As mentioned previously, all studies determined CIMP status based on their own set of genes, even though we saw a few recurrent genes such as GSTP1 and p16. This is partly due to the technology limitation in the past as most studies used MSP for methylation profiling. With genome-wide profiling methods become more available, it is a matter of time that we can soon ascertain the methylation markers that make up Hep-CIMP.

### DNA methylation as potential blood biomarker

HCC is associated with high mortality rate mainly due to late diagnosis [1]. Therefore, there is an urgent need to identify promising tool that could diagnose the disease early or be served as surveillance for patients at risk. DNA methylation profile derived from blood samples could potentially be such biomarker. The attempt to identify methylation marker in blood dated back as early as 2003, where Wong et al. used quantitative MSP to measure the methylation status of p16 in 29 HCC patients [103]. However, they did not perform any clinical association with their data. In fact, many studies successfully measure the aberrant methylation level of a marker gene in blood but did not associate it with clinicopathological parameters. These studies can be found in Table 5.

**Table 5:** Methylation studies on DNA extracted from HCC blood samples

**With clinicopathological correlation**						
Marker genes	HCC patients (n)	Clinicopathological correlation	Validation method	Samples used for DNA extraction	Publication	Year
DAPK, p16	64	AFP level	MSP	Serum	Lin et al. [104]	2005
RASSF1A	40	Tumor size	MSP	Plasma	Yeo et al. [106]	2005
LINE-1	85	OS, tumor size	COBRA	Serum	Tangkijvanich et al. [109]	2007
RASSF1A	85	DFS	Methylscreen	Serum	Chan et al. [107]	2008
CCND2	70	DFS	qMSP	Serum	Tsutsui et al. [105]	2010
APC, DKK, DLC1, CDH1, RUNX3, SFRP1, WIF1	108	CIMP + associated with gender, HBV infection, AFP level, tumor stage, DFS	MSP	Plasma	Liu et al. [101]	2011
APC, GSTP1, RASSF1A, SFRP1	72	OS (APC, RASSF1A)	Methylscreen	Plasma	Huang et al. [108]	2011
LINE-1	305	Increased risk of HCC	Pyrosequencing	White blood cells	Wu et al. [110]	2012
IGFBP7	136	Vascular invasion	MSP	Serum	Li et al. [115]	2013
XPO4	44	AFP level	MSP	PBMC	Zhang et al. [114]	2013
TFPI2	43	Tumor stage	MSP	Serum	Sun et al. [113]	2013
APC	23	Portal vein thrombosis	qMSP	Serum	Nishida et al. [112]	2013
**Without clinicopathological correlation**						
Marker genes	HCC patients (n)	Clinicopathological correlation	Validation method	Samples used for DNA extraction	Publication	Year
p16	29	NIL	qMSP	Serum and buffy coat	Wong et al. [103]	2003
p16	46	NIL	MSP	Serum	Chu et al. [121]	2004
GSTP1	32	NIL	MSP	Serum	Wang et al. [122]	2006
CDH1, p16, RASSF1A, RUNX3	8	NIL	MSP	Serum	Tan et al. [123]	2007
p16, p15, RASSF1A	50	NIL	MSP	Serum	Zhang et al. [124]	2007
GSTP1, RASSF1A	26	NIL	MSP	Serum	Chang et al. [125]	2008
RASSF1A	35	NIL	MSP	Serum	Hu et al. [126]	2010
APC, CDH1, FHIT, p15, p16	28	NIL	MSP	Plasma	Iyer et al. [127]	2010
DBX2, THY1	31	NIL	Bisulfite seq	PBMC	Zhang et al. [41]	2013

*AFP*, Alpha fetoprotein; *COBRA*, Combined Bisulfite Restriction Analysis; *DFS*, Disease free survival; *HBV*, Hepatitis B virus; *MSP*, Methylation-specific PCR; *qMSP*, Quantitative MSP; *OS*, Overall survival; *PBMC*, Peripheral blood mononuclear cells; *Seq*, Sequencing.

In this section, we will only highlight reports with significant clinical association. As shown in Table 5, Lin et al. showed that among 64 patients, about 77% of them have p16 methylation and 41% of them have DAPK methylation. Both markers were associated with AFP levels but no other parameters [104]. Tsutsui et al. found that the serum of 39 out of 70 patients were positive for methylated CCND2 gene. Patients of this group had shorter disease free survival [105].

Yeo et al. used MSP and found that 17 out of 40 patients’ plasma (42.5%) had RASSF1A hypermethylation and their methylation status was associated with tumor size [106]. Chan et al. used another method, methylation-sensitive restriction enzyme-mediated real-time PCR system, to detect RASSF1A methylation status in 85 HCC sera. They found that 93% of them have hypermethylation and their methylated status was associated with shorter disease free survival and time-to-occurrence for HCC [107]. Using similar detection method, Huang et al. also showed that RASSF1A gene in 72 patients’ blood was hypermethylated compared to normal controls and that its methylation level was associated with poorer overall survival [108].

Tangkijvanich et al. used Combined Bisulfite Restriction Analysis (COBRA) method to measure the hypomethylation of LINE-1 in 85 patients’ sera. They reported that hypomethylation of LINE-1 was associated with HBV infection, larger tumor size and more advance disease stage [109]. Their study was further validated by Wu et al., where they used pyrosequencing to determine the methylation level of LINE-1 in 305 patients’ white blood cell DNA [110]. They used logistic regression model to show that hypomethylation of LINE-1 increased overall risk of developing HCC. Recently, Gao et al. also reported that LINE-1 was hypomethylated in 71 HCC tissues and was associated with poorer prognosis [111]. These are few studies that showed hypomethylation instead of hypermethylation as potential prognostic biomarker.

Following the availability of genome-wide methylation profile, we also saw a sudden surge of methylation studies based on patients’ sera. Within year 2013, four studies reported prognostic value of four different hypermethylated genes. Briefly, Nishida et al. performed quantitative MSP and showed that APC was more methylated in 23 HCC sera compared to healthy volunteers. They also showed that patients with higher APC methylation were associated with portal vein thrombosis [112]. Sun et al. detected TFPI2 to be more methylated in 43 HCC sera and its level was associated with TNM stage [113]. Zhang et al. on the other hand found XPO4 to be frequently methylated in 44 patients’ peripheral blood mononuclear cells. Their data indicated that higher XPO4 methylation was associated with higher AFP level [114]. Lastly, Li et al. discovered that in HBV-associated HCC, IGFBP7 was more methylated compared to chronic hepatitis B patients and normal controls. Also, its methylation status was associated with vascular invasion in HCC [115].

Clearly, methylation of marker gene in HCC blood DNA has potential prognostic value as shown by its association with clinicopathological data. However, most current studies drawn conclusion from a retrospective cohort. In order to translate these markers into actual clinical use, proper prospective studies and validation method are required.

## Conclusions

Many genome wide methylation studies have confirmed that HCC has distinct methylation profile (Table 2), and some even showed that it is associated with different etiological factors such as HBV infection and alcohol consumption. Undeniably, the availability of these genome wide data has allowed the discovery of many novel genes with aberrant methylation, especially in recent years. As shown in Additional file 1: Table S1, apart from the commonly studied genes mentioned in this review, there is plethora of genes that were differentially methylated and associated with clinicopathological data. Future studies need to focus on collating current available data, shortlisting potential methylation markers by conducting proper validation method [116,117] and defining well-characterized CIMP status of HCC. It is hoped that emerging methylation markers can be used as diagnostic or prognostic marker for HCC in near future.

## Abbreviations

AFP: Alpha fetoprotein; CIMP: CpG island methylator phenotype; COBRA: Combined bisulfite restriction analysis; DFS: Disease free survival; DMH-chip: Differential methylation hybridization on microarray; DNMT: DNA methyltransferase; HBV: Hepatitis B virus; HBx: Hepatitis B virus X protein; HCC: Hepatocellular Carcinoma; HCV: Hepatitis C virus; MCAM: Methylated CpG island amplification microarray; MeDIP-Chip: Methylated DNA immunoprecipitation microarray; MSP: Methylation-specific PCR; OS: Overall survival; PBMC: Peripheral blood mononuclear cells; PCR: Polymerase chain reaction; QMSP: Quantitative MSP; RFS: Recurrence free survival.

## Competing interests

All authors declare that they have no conflicts of interests.

## Authors’ contributions

CGL and W-C researched data and wrote the manuscript. Both authors read and approved the final manuscript.

## Supplementary Material

Additional file 1: Table S1 List of methylation studies on HCC from year 2003–2013. Click here for file
