# Supplementary material for: DNA methylation: potential biomarker in Hepatocellular Carcinoma
Source: Biomark Res. 2014 Mar 17;2:5. doi: 10.1186/2050-7771-2-5 (PMC4022334; doi:10.1186/2050-7771-2-5)
Supplement: Additional file 1: Table S1 — List of methylation studies on HCC from year 2003–2013. [file 2050-7771-2-5-S1.pdf]

Supplementary table 1. List of methylation studies on HCC from year 2003-2013.

Type of study	Validated gene name	Publication	Year	hyper / hypo	HCC patient (n)	Clinicopathological correlation	Validation method	Sample
Gene based	GLS2	Zhang, J. et al.	2013	hyper	20	No	MSP	tissue
Genome-wide	ERG, HOXA9	Hou, X. et al.	2013	hyper	45	Yes	Bisulfite sequencing	tissue
Genome-wide	NEFH, SMPD3	Revill, K. et al.	2013	hyper	71	Yes	Pyrosequencing	tissue
Gene based	DACH1	Zhu, H. et al.	2013	hyper	55	Yes	MSP, qMSP	tissue
Gene based	RB, P14, and INK4 gene family	Zhang, J.-C. et al.	2013	hyper	64	Yes	MSP	tissue
Genome-wide	No specific gene validated	Allen Chan,K.C. et al.	2013	hypo	26	No	Bisulfite massively parallel sequencing	plasma
Gene based	PMS2	Ozer, O. et al.	2013	hyper	56	Yes	Multiplex ligated probe amplification (MLPA)	FFPE
Gene based	DNM3	Inokawa, Y. et al.	2013	hyper	48	Yes, based on gene expression.	MSP	tissue
Gene based	IGFBP7	Li, F. et al.	2013	hyper	136	Yes	MSP	serum
Gene based	DACH1	Zhu, H. et al.	2013	hyper	55	Yes, based on gene expression.	MSP, Methylscreen	tissue
Gene based	BTG3	Lv, Z. et al. et al.	2013	hyper	141	Yes, based on gene expression.	MSP	FFPE
Gene based	DCDC2	Inokawa, Y. et al.	2013	hyper	48	Yes	MSP	tissue
Gene based	RASSF1A	Xu, B. et al. et al.	2013	hyper	87	Yes	Methylight	tissue
Gene based	SOCS1	Zhang, X. et al.	2013	hyper	116	Yes	Methylscreen	tissue
Gene based	LINE-1	Gao, X.-D. et al.	2013	hypo	172	Yes	qMSP	tissue
Gene based	GPM6B, MAGEA12, FCRL1	Stefanska, B. et al.	2013	hypo	16	Yes	HRM	tissue
Gene based	PTPRO	Hsu, S.-H. et al.	2013	hyper	24	No	MassARRAY	tissue
Gene based	XPO4	Zhang, F. et al.	2013	hyper	44	Yes	MSP	peripheral blood mononuclear cells (PBMCs)
Gene based	ESR1	Hishida, M. et al.	2013	hyper	48	Yes, based on gene expression.	MSP	tissue
Genome-wide	DBX2, THY1	Zhang, P. et al.	2013	hyper	6 (discovery); 31(validation)	No	Infinium450k; bisulfite sequencing	peripheral blood mononuclear cells (PBMCs)
Gene based	ABCB6	Tsunedomi, R. et al.	2013	hyper	25	Yes	qMSP	tissue
Gene based	Klotho	Xie, B. et al.	2013	hyper	64	Yes	MSP	tissue
Gene based	APC, AXIN2	Liu, S. et al.	2013	hyper	80	Yes, based on gene expression.	Methylscreen	tissue
Gene based	PCDH10	Fang, S. et al.	2013	hyper	50	Yes	MSP	tissue
Gene based	ZAR1	Takagi, K. et al.	2013	hyper	88	Yes	MassARRAY	tissue
Gene based	TFPI2	Sun, F-K. et al.	2013	hyper	43	Yes	MSP	serum
Gene based	MAT1A	Zhang, J. et al.	2013	hyper	78	Yes	MSP	tissue
Gene based	DACT2	Gao, S. et al.	2013	hyper	30	Yes	MSP	tissue
Gene based	SOX1	Shih, Y.-L. et al.	2013	hyper	54	No	MSP	tissue
Genome-wide	BMP4, GSTP1, RASSF1A, ARHGAP8, TACSTD2, DLGAP, GPR35	Song, M-A. et al.	2013	hyper	27	No	Infinium450k,pyrosequencing	tissue
Gene based	ASS	Wu, L. et al.	2013	hyper	30	No	MSP	tissue
Gene based	APC	Nishida, N. et al.	2013	hyper	23	Yes	qMSP	serum
Gene based	APC, P73, P14, MGMT	Zekri, A.-R.N. et al.	2013	hyper	31	Yes	MSP	tissue
Gene based	MUC2	Ling, Y. et al.	2013	hyper	74	Yes	MSP	tissue
Gene based	BLU,FHIT	Zhang, X. et al.	2013	hyper	48	Yes	MSP	tissue
Genome-wide	No specific gene validated	Shen, J. et al.	2013	hyper	66	No	Infinium450k; bisulfite sequencing	tissue
Gene based	SOCS1	Saelee, P. et al.	2012	hyper	29	Yes	MSP	tissue
Gene based	MT1M	Mao, J. et al.	2012	hyper	8	Yes, based on gene expression.	MSP	tissue
Gene based	SOX1	Tsao, C-M et al.	2012	hyper	60	No	qMSP	tissue
Gene based	COX2	Fernández-Alvarez, A. et al.	2012	hyper	23	No	Pyrosequencing	tissue
Gene based	TOB1	Lin, S. et al.	2012	hyper	33	No	MassARRAY	tissue
Gene based	PER3	Neumann, O. et al.	2012	hyper	63	No	Infinium27K, pyrosequencing	tissue
Gene based	KLHL35, PAX5, PENK, SPDYA -hypermethylaiton, LINE-1 - Hypomethylation	Shitani, M. et al.	2012	hyper	57	Yes	MCAM, pyrosequencing	tissue
Gene based	HIC1, GSTP1, SOCS1, RASSF1, CDKN2A, APC, RUNX3, PRDM2	Nishida, N. et al.	2012	hyper	177	Yes	Methylight	tissue
Gene based	SFRP1	Wu, Y. et al.	2012	hyper	11	No	MSP	tissue
Gene based	LINE-1	Wu, H.-C. et al.	2012	hypo	305	Yes	Pyrosequencing	white blood cells
Gene based	MZB1	Matsumura, S. et al.	2012	hyper	162	Yes, based on gene expression.	MeDIP-Chip	tissue
Gene based	HIST1H2AE	Jung, N. et al.	2012	hyper	10	Yes	MSP	tissue
Gene based	SALL3	Yang, X.-X et al.	2012	hyper	38	No	MassARRAY	tissue
Genome-wide	CDKL2, STEAP4, HIST1H3G, CDKN2A, ZNF154	Shen, J. et al.	2012	hyper	62 (discovery), 42 (validation)	No	Infinium27K, pyrosequencing	tissue
Gene based	SPARC	Zhang, Y. et al.	2012	hyper	60	Yes	MSP	tissue
Gene based	RASSF1A, CHFR, GSTP1, MGMT	Li, Z. et al.	2012	hyper	70	Yes	MSP	tissue
Gene based	SFRP1	Kaur, P. et al.	2012	hyper	138	No	Pyrosequencing	tissue
Genome-wide	LOC55908, CELSR1, CRMP1, GNRH2, ALOX12 and ANGPTL7 -hypermethylation, SPRR3 and TNFSF15- hypomethylation	Ammerpohl, O. et al.	2012	hyper, hypo	13	No	Infinium27k	tissue
Gene based	AKAP12	Hayashi, M. et al.	2012	hyper	48	Yes	MSP	tissue
Gene based	RECK	Zhang, C. et al.	2012	hyper	74	Yes, based on gene expression.	MSP	tissue
Gene based	P16 , CCND2	Wang, Y. et al.	2012	hyper	118	Yes	Methylscreen	tissue
Gene based	SLC22A1	Schaeffeler, E. et al.	2011	hyper	22	No	MassARRAY	tissue
Gene based	BLMH	Okamura, Y. et al.	2011	hyper	48	No	Infinium27K, MSP	tissue
Gene based	APC, GSTP1,RASSF1A, SFRP1	Huang, Z.-H. et al.	2011	hyper	72	Yes	Methylscreen	serum
Gene based	APC, WIF-1, RUNX-3, DLC-1, SFRP-1, DKK , CDH1	Liu, J.-B. et al.	2011	hyper	108	Yes	MSP	plasma and tissue
Gene based	PRDM5	Shu, X.-s et al.	2011	hyper	46	No	MSP	tissue
Gene based	APC	Jain, S. et al.	2011	hyper	58	Yes	qMSP	tissue
Genome-wide	No specific gene validated	Yang, J.D. et al.	2011	hyper	62	Yes	Infinium27k	tissue
Gene based	FBP1	Chen, M. et al.	2011	hyper	10	No	MSP	tissue
Gene based	OCT6	Sun, J.-Z. et al.	2011	hyper	38	No	MassARRAY	tissue
Gene based	30 regions	Nagashio, R. et al.	2011	hyper, hypo	34	Yes	Pyorsequencing	tissue
Genome-wide	MBD2	Stefanska, B. et al.	2011	hypo	11	No	MeDIP-Chip, pyrosequencing	tissue
Gene based	APC, CDKN2A, GSTP1, RASSF1A, SFRP1, RUNX3	Hua, D. et al.	2011	hyper	47	No	Methylscreen	tissue
Gene based	FBLN1	Kanda, M. et al.	2011	hyper	48	Yes	MSP	tissue
Gene based	SIP1	Acun, T. et al.	2011	hyper	39	No	COBRA	tissue
Gene based	CD147	Kong, L.-M. et al.	2011	hypo	54	Yes	Bisulfite sequencing	tissue
Genome-wide	WNK2, EMILIN2, TLX3, TM6SF1, TRIM58, HIST1H4F, GRASP	Tao, R. et al.	2011	hyper	5	No	Infinium27K, COBRA, bisulfite sequencing	tissue
Gene based	APC, RASSF1A, SOCS1	Um, T.-H. et al.	2011	hyper	46	Yes	MethyLight	tissue
Gene based	CADM1	Zhang, W et al.	2011	hyper	82	Yes	COBRA	tissue
Gene based	RASSF1A, GSTP1, CHRNA3, DOK1	Lambert, M.-P. et al.	2011	hyper	166	Yes	Pyrosequencing	tissue
Gene based	Subtelomeric DNA	Oh, B.-K. et al.	2011	hyper	32	Yes	MSP	tissue
Gene based	RELN	Okamura, Y. et al.	2011	hyper	48	Yes, based on gene expression.	MSP	tissue
Gene based	RASSF1A	Saelee, P. et al.	2010	hyper	29	No	MSP	tissue
Genome-wide	MYLK, FLT1, CDKN1C, TAp73	Hernandez-Vargas, H. et al.	2010	hyper	30	Yes	Beadarray, pyrosequencing	tissue
Gene based	AKAP12	Goeppert, B. et al.	2010	hyper	16	No	MassARRAY	tissue
Gene based	RIZ1	Zhang, C. et al.	2010	hyper	48	No	MSP	tissue
Gene based	P16, RASSF1A, APC, GSTP1, RIZ1	Formeister, E.J. et al.	2010	hyper	43	Yes	MSP	tissue
Gene based	P16, CDH1, GSTP1, DAPK, XAF1, SOCS1, SYK	Wu, L.-M. et al.	2010	hyper	65	Yes	MSP	tissue
Gene based	TNFRSF10C, HOXA9, NPY, IRF5	Shin, S.H. et al.	2010	hyper	50	No	MSP	tissue
Gene based	P14, P15, P16 ,P73, GSTP1, MGMT,RARβ, SOCS1, OPCML	Li, B. et al.	2010	hyper	115	Yes	MSP	tissue
Gene based	SOCS1	Chu, P.-Y et al.	2010	hyper	46	Yes	MSP	tissue
Gene based	CDKN2A	Csepregi, A. et al.	2010	hyper	85	No	MethyLight	tissue
Genome-wide	DUSP4, NPR1, CYP24A1	Deng, Y.-B. et al.	2010	hyper	61	Yes	MeDIP-Chip, Massarray	tissue
Gene based	TTP	Sohn, B.H et al.	2010	hyper	24	No	Pyrosequencing	tissue
Gene based	APC, CCND2, CDKN2A, GSTP1, HOXA9, RASSF1, RUNX, SFRP1	Feng, Q. et al.	2010	hyper	65	Yes	Methylight	FFPE
Gene based	APC	Archer, K.J. et al.	2010	hyper	20	No	Beadarray, methylight	tissue
Gene based	CCND2	Tsutsui, M. et al.	2010	hyper	70	Yes	qMSP	serum
Gene based	LIFR	Okamura, Y. et al.	2010	hyper	48	No	MSP	tissue
Gene based	CASP8 and Survivin	Cho, S. et al.	2010	hyper	73	Yes, based on gene expression.	MSP	tissue
Gene based	RASSF1A	Hu, L. et al.	2010	hyper	35	No	MSP	tissue and serum
Gene based	EFEMP1	Nomoto, S. et al.	2010	hyper	48	Yes	MSP	tissue
Gene based	PLK2, PLK3, PLK4	Pellegrino, R. et al.	2010	hyper	75	No	MSP	tissue
Gene based	DKK3	Yang, B. et al.	2010	hyper	50	Yes	qMSP	tissue
Gene based	APC, FHIT, P15, P16,CDH1	Iyer, P. et al.	2010	hyper	28	No	MSP	tissue and plasma
Gene based	P14, P15, P16, P21, SYK, TIMP-3, WT1, CDH1, RASSF1A, RB1	Cheng, Y. et al.	2010	hyper	60	Yes	MSP	tissue
Genome-wide	41 BAC regions	Arai, E. et al.	2009	hyper	126	Yes	Bacterial artificial chromosome (BAC) array-based methylated CpG island amplification	tissue
Gene based	KLK10 and OXGR1	Lu, C.-Y. et al.	2009	hyper	21 (discovery), 49 (validation)	Yes	Differential methylation hybridization and a CpG microarray platform; MSP	tissue
Gene based	MT1G	Kanda, M. et al.	2009	hyper	48	No	MSP	tissue
Gene based	RASSF1A, CCND2,SPINT2, RUNX3, GSTP1, APC, CFTR	Moribe, T. et al.	2009	hyper	20	No	qMSP, pyrosequencing	tissue
Gene based	APC, RASSF1A, DAPK, SOCS1, GSTP1, RIZ1, P16, MGMT	Lou, C. et al.	2009	hyper	60	Yes	MSP	tissue
Gene based	SPINT2	Tung, E.K.-K. et al.	2009	hyper	50	No	MSP	tissue
Gene based	HINT1	Zhang, Y et al.	2009	hyper	40	No	MSP	tissue
Gene based	CRABP1, SYK	Lee, H.S. et al.	2009	hyper	20	Yes	Methylight	tissue
Gene based	SLIT2	Jin, J. et al.	2009	hyper	54	Yes	MSP	tissue
Gene based	LINE-1	Kim, M.-J. et al.	2009	hypo	57	Yes	pyrosequencing	tissue
Gene based	RIZ1	Piao, G.H. et al.	2008	hyper	39	No	MSP	tissue
Gene based	BASP1, SRD5A2	Moribe, T. et al.	2008	hyper	40	No	MSP, pyrosequencing	tissue
Gene based	CDH1	Lim, S.-O. et al.	2008	hyper	42	No	MSP	tissue
Gene based	XAF1	Zhang, F. et al.	2008	hyper	65	Yes	MSP	tissue
Gene based	SOCS1	Ko, E. et al.	2008	hyper	284	Yes	MSP	tissue
Gene based	Tip30	Lu, B. et al.	2008	hyper	59	Yes	MSP	tissue
Gene based	P14	Zhang, C. et al.	2008	hyper	30	Yes	MSP	tissue
Genome-wide	RASSF1A, P16, TBX4, MMP14, GNA14, SLC16A5, CCNA1	Gao, W. et al.	2008	hyper hypo	10 (MCAM), 38 (validation)	No	MCAM,pyrosequencing	tissue
Gene based	GSTP1, RASSF1A	Chang, H. et al.	2008	hyper	37 (tissue), 26(plasma)	No	MSP	tissue and serum
Gene based	P16	Ko, E. et al.	2008	hyper	265	Yes	MSP	tissue
Gene based	P21, P15, P16, WTI, E2F1	Zhang, C. et al.	2008	hyper	120	Yes	MSP	tissue
Gene based	RASSF1A	Chan, K.C.A. et al.	2008	hyper	85	Yes	Methylscreen	tissue and serum
Gene based	UCHL1	Yu, J. et al.	2008	hyper	27	No	MSP	tissue
Gene based	GSTP1, P16	Harder, J. et al.	2008	hyper	34	No	MSP	tissue
Gene based	HHIP	Tada, M. et al.	2008	hyper	36	No	Methylight	tissue
Gene based	NDRG2	Dong, C.L. et al.	2008	hyper	9	Yes, based on gene expression.	MSP	tissue
Gene based	SFRP	Takagi, H. et al.	2008	hyper	19	No	MSP	tissue
Gene based	DLEC1	Qiu, G.-H. et al.	2008	hyper	68	Yes	MSP	tissue
Gene based	HIC1, CASP8, GSTP1, SOCS1, RASSF1A, P16, APC, RUNX3, RIZ1, SFRP2, CDH1, COX2, CACNA1G, RASSF2,Reprimo, DCC, and 3 MINT loci	Nishida, N. et al.	2008	hyper	77	Yes	COBRA	tissue
Gene based	P16, GSTP1, APC,RIZ1,SFRP1,SFRP2,SFRP5,RUNX3, SOCS1	Nomoto, S. et al.	2007	hyper	19	No	MSP	tissue
Gene based	RUNX3, P16, RASSF1A and CDH1	Tan, S.-H. et al.	2007	hyper	8	No	MSP	serum
Gene based	P16	Kondo, Y. et al.	2007	hyper	23	No	MSP	tissue
Gene based	P16, GSTP1	Su, P.-F. et al.	2007	hyper	58	Yes	MSP	tissue
Gene based	genome-wide hypomethylation, BNIP3, BNIP3L, IGFBP3, and EGLN2; SPRY1 and -2, DAB2, and SOCS4 and -5	Calvisi, D.F. et al.	2007	hyper	60	Yes	MSP, COBRA	tissue
Gene based	P16, GSTP1, IGF2	Dejeux, E. et al.	2007	hyper	27	No	Pyrosequencing	tissue
Gene based	SFRP1, SFRP2, SFRP5	Shih, Y.-L. et al.	2007	hyper	54	No	MSP	tissue
Gene based	SFRP1	Huang, J. et al.	2007	hyper	2	No	Bisulfite sequencing	tissue
Gene based	P16, CDH1, RASSF1A	Oh, B.-K. et al.	2007	hyper	25	No	MSP	tissue
Gene based	RASSF1A, CCND2, P16, GSTP1, SOCS-1, APC	Lehmann, U. et al.	2007	hyper	17	No	qMSP	tissue
Gene based	P16	Zhu, R. et al.	2007	hyper	23	Yes	MSP	tissue
Gene based	SOCS1, CASP8, RUNX3, HIC-1, GSTP1, P16, RASSF1A, RASSF2, APC, RIZ1, COX2, CACNA1G, Reprimo, SFRP2	Nishida, N. et al.	2007	hyper	81	Yes	COBRA	tissue
Gene based	TFPI-2	Wong, C.-M. et al.	2007	hyper	34	No	MSP	tissue
Gene based	RASSF1A, P16, P15	Zhang, Y.-J. et al.	2007	hyper	50	No	MSP	blood
Gene based	hDAB2IPA	Qiu, G.-H. et al.	2007	hyper	53	No	MSP	tissue
Gene based	LINE-1	Tangkijvanich, P. et al.	2007	hypo	85	Yes	COBRA	serum
Gene based	P14, P15, P16, ER, RASSF1A, WT1, and c-Myc	Zhang, C. et al.	2007	hyper	50	Yes	MSP	tissue
Gene based	SYK	Yuan, Y. et al.	2006	hyper	124	Yes	MSP	tissue
Gene based	P16	Zhang, Y.-J. et al.	2006	hyper	40	No	MSP	tissue
Gene based	SFRP1	Shih, Y.-L. et al.	2006	hyper	54	No	MSP	tissue
Gene based	IGF-II	Tang, S.H. et al.	2006	hypo	34	Yes	MSP	tissue
Gene based	P16	Jicai, Z. et al.	2006	hyper	44	Yes	MSP	tissue

Gene based	NORE1B, RASSF1A	Macheiner, D. et al.	2006	hyper	40	No	MSP	tissue
Gene based	ZHX2	Lv, Z. et al.	2006	hyper	32	Yes	MSP	tissue
Gene based	RASSF1A	Di Gioia et al.	2006	hyper	26	Yes	MSP	tissue
Gene based	GSTP1	Wang, J. et al.	2006	hyper	26 (tissue), 32 (serum)	No	MSP	tissue and serum
Gene based	MINT31, P16, cox2,GSTP1,RASSF1A,CDH1,APC	Katoh, H. et al.	2006	hyper	60	Yes	MSP	tissue
Gene based	MAGEA1, MAGEA3	Qiu, G. et al.	2006	hyper	34	No	MSP	FFPE
Gene based	P16, RASSF1A	Park, H-J. et al.	2006	hyper	27	No	MSP	tissue
Gene based	P16	Yang, B. et al.	2005	hyper	20	No	MSP	tissue
Gene based	P16	Fukai, K. et al.	2005	hyper	39	No	MSP	tissue
Gene based	P16, DAPK	Lin, Q. et al.	2005	hyper	64	Yes	MSP	serum
Gene based	SOCS-3	Niwa, Y. et al.	2005	hyper	18	No	MSP	tissue
Gene based	RUNX3,	Park, W.S. et al.	2005	hyper	73	No	MSP	tissue
Gene based	RASSF1A, SEMA3B	Tischoff, I. et al.	2005	hyper	35	No	MSP	tissue
Gene based	RASSF1A, cyclinD2, P16, GSTP1, APC, RIZ1, SOCS1	Lehmann, U. et al.	2005	hyper	41	No	qMSP	FFPE
Gene based	GSTP1	Zhang, Y.-J. et al.	2005	hyper	83	Yes	MSP	tissue
Gene based	CDH1	Ghee, Y.K. et al.	2005	hyper	32	Yes	MSP	tissue
Gene based	GSTP1, NOO1	Tada, M. et al.	2005	hyper	44	No	MSP	tissue
Gene based	RASSF1A	Yeo, W. et al.	2005	hyper	40	Yes	MSP	tissue and plasma
Gene based	HDRP1	Yau, T.-O. et al.	2005	hyper	43	No	MSP	tissue
Gene based	P16	Li, X. et al.	2004	hyper	50	Yes	MSP	tissue
Gene based	GADD45B	Qiu, W. et al.	2004	hyper	8	No	MSP	tissue
Gene based	ASC	Kubo, T. et al.	2004	hyper	17	No	MSP	tissue
Gene based	P16, P15	Qin, Y. et al.	2004	hyper	20	Yes	MSP	tissue
Gene based	P16	Narimatsu, T. et al.	2004	hyper	51	No	MSP	tissue
Gene based	P16,P14, GSTP1	Anzola, M. et al.	2004	hyper	30	No	MSP	tissue
Gene based	P16	Chu, H.J. et al.	2004	hyper	46	No	MSP	serum
Gene based	SPINT2	Fukai, K. et al.	2003	hyper	26	No	MSP	tissue
Gene based	DLC-1	Wong, C.-M. et al.	2003	hyper	25	No	MSP	tissue
Gene based	SOCS-1	Okochi, O. et al.	2003	hyper	50	Yes	MSP	tissue
Gene based	ABO, AR, CSPG2, cyclin a1, DBCCR1, GALR2, IRF7, MGMT, MT1A, MYOD1, OCT6, p57KIP2, P73, WT1, P16, and demethylation of the MAGEA1	Yu, J. et al.	2003	hyper	28	Yes	MSP	tissue
Gene based	APC, GSTP1, RASSF1A, P16, COX-2, CDH1	Lee, S. et al.	2003	hyper	60	Yes	MSP	tissue
Gene based	SOCS-1, GSTP, APC, CDH1, P15	Yang, B. et al.	2003	hyper	51	Yes	MSP	tissue
Gene based	RASSF1A	Zhong, S. et al.	2003	hyper	43	No	COBRA	tissue
Gene based	P16	Matsuda, Y. et al.	2003	hyper	12	Yes, based on gene expression.	MSP	tissue
Gene based	RASSF1A, P16	Schagdarsurengin, U., et al.	2003	hyper	15	No	MSP	tissue
Gene based	P16	Wong, I.H.N. et al.	2003	hyper	29	Yes	qMSP	serum and buffy coat
Gene based	MGMT	Matsukura, S. et al.	2003	hyper	46	No	Bisulfite sequencing	tissue
Gene based	P16	Shim, Y.-H. et al.	2003	hyper	18	Yes	MSP	FFPE
Gene based	MGMT	Zhang, Y.-J. et al.	2003	hyper	83	No	MSP	tissue
